# Symptoms affecting the development of diabetes: analysis of risk factors with data mining

**DOI:** 10.1186/s12911-025-03159-5

**Published:** 2025-08-27

**Authors:** Ali Vasfi Aglarci, Feridun Karakurt

**Affiliations:** 1 Department of Biostatistics, Faculty of Medicine, Kastamonu University, Kastamonu, Turkey; 2 Department of Internal Medicine, Faculty of Medicine, Necmettin Erbakan University, Konya, Turkey

**Keywords:** Diabetes prediction, Association analysis, Apriori algorithm, Early detection, Data mining, Symptom analysis

## Abstract

**Objective:**

Diabetes is one of the most common chronic health threats worldwide. Early detection of diabetes is difficult and diagnosis and treatment processes can be costly. Data mining techniques offer powerful tools for predictive analysis and knowledge extraction from large data sets. This study aims to identify symptoms that collectively influence the development of diabetes by data mining and identify risk parameters for early detection.

**Materials and methods:**

The study uses a dataset of 520 patient records collected from Sylhet Diabetes Hospital in Sylhet, Bangladesh. This dataset is based on real-world data from the UCI Machine Learning Repository. The Apriori algorithm, which is widely used in data mining, was applied to analyze the symptoms associated with diabetes using association analysis. The algorithm analyzed the relationships between symptoms based on support, confidence and lift values.

**Results:**

The analysis identified eight key symptoms that significantly contribute to diabetes risk when they occur together: gender, polyuria, polydipsia, sudden weight loss, weakness, blurred vision, partial paresis and obesity. The co-occurrence of these symptoms increases the likelihood of developing diabetes by 1.63 times. These findings emphasize the importance of assessing symptoms collectively rather than in isolation.

**Conclusion:**

The results of the study emphasize the importance of individuals at risk of diabetes and healthcare professionals to monitor these symptoms and take necessary precautions. The study shows that association rule mining, especially the Apriori algorithm, is a valuable tool for identifying symptom associations and facilitating early diabetes detection. The findings will contribute to early detection of diabetes and prevention of complications related to the disease through simple symptom analysis.

## Introduction

Diabetes is a chronic disease that affects individual, economic and social well-being and can lead to many serious long-term complications such as cardiovascular disease, stroke, kidney failure, heart attack, peripheral arterial disease, blood vessels and nerves [1–4]. Urbanization, unhealthy lifestyles and an ageing population are the main reasons for the steady increase in the prevalence of diabetes [5]. In 2021, approximately 537 million adults worldwide will be living with diabetes, which is considered a global health crisis [1]. It is estimated that 1.6 million deaths occur directly due to diabetes and the number of people with diabetes will reach 642 million by 2040 [6, 7]. The increase in the number of people with diabetes and the rise in deaths related to this condition constitute an alarming trend [4]. The overall mortality risk of people with diabetes is at least twice as high compared to their peers without diabetes [8]. In this context, the World Health Organization (WHO) has identified diabetes as one of the most common chronic life threats [9]. It is also estimated that approximately 50% of individuals with diabetes do not receive a diagnosis of their disease because the symptoms become apparent [9]. The signs and symptoms of diabetes are often overlooked by many people due to the chronic progression of the disease. Unlike other diseases, the effects of hyperglycemia are not immediately apparent, so people do not perceive it as a serious health problem [10]. Around 80% of people with diabetes worldwide are reported to live in low- and middle-income countries with inadequate health infrastructure. It is also reported that diabetes is more prevalent in the elderly population [9].

Individuals with diabetes are at high risk of developing health problems such as cardiopathy, kidney impairment, stroke, nerve damage and eye damage. Early detection of diabetes is difficult and diagnosis and treatment processes can be costly [11]. Globally, diabetes has led to health expenditures of approximately 966 billion dollars and is expected to exceed 1,054 billion dollars by 2045 [5, 12–14]. Early detection and treatment can prevent or significantly reduce the severity of these health problems. Studies have shown that diseases detected early are more likely to be cured than those detected late [15]. Rapid diagnosis and treatment can reduce the costs and duration of health care [9]. Recognizing early symptoms can help to rapidly control the disease and prevent complications.

In this context, researchers have started to utilize data mining techniques using data on parameters that may lead to diabetes. The fields of data mining and machine learning are powerful in performing predictive analysis and knowledge extraction from large data sets [16, 17]. Using these methods to predict early intervention by extracting information from data sets can lead to more robust decisions for the rapid diagnosis of diabetes [8]. Data mining and machine learning have developed in recent years as reliable and supportive tools in the medical field [18, 19]. In the literature, many studies have been conducted on diabetes diagnosis using data mining and machine learning methods [15, 18, 20–23]. However, it is seen that different parameters are used in these studies and blood test data are among the parameters in some studies. It is known that obtaining blood test data is time and cost consuming [24]. Moreover, in such studies, the effect of each parameter is evaluated individually and the effect of the parameters together is ignored. In some cases, a high frequency of symptoms alone is not significant, but in combination they may trigger the disease or pose a risk. Conversely, when some symptoms occur at a low frequency, they may combine with other symptoms to trigger the disease. In line with this need, unlike existing studies, the evaluation of the combined effect of symptoms for early detection of diabetes is addressed. Apriori algorithm was used for association analysis from data mining methods. To the best of our knowledge, there is no study in the literature that examines the association effect of symptoms on diabetes. The Apriori algorithm is a simple and common data mining algorithm that extracts association rules from data sets and is often used in grocery basket analysis [25]; however, it has limited use in the health field for evaluating the joint effects of parameters affecting diseases.

The aim of this study is to identify the co-occurring symptoms of diabetes and to identify easy-to-access parameters other than blood tests that may pose a risk for early diagnosis of diabetes. In addition, this study aims to contribute to the United Nations Sustainable Development Goals of “health and quality of life”.

## Literature

There are many studies in the literature on the diagnosis and identification of diabetes through data mining. Looking at these studies;

A model has been developed for the timely prediction and severity level of diabetes using Machine Learning (ML) approaches. A hybrid model was developed using traditional models such as Artificial Neural Network (ANN), Support Vector Machine (SVM), Random Forest (RF), Decision Tree (DT), and AdaBoost. An accuracy of 97% for the initial classification and 79% for the validation dataset, and 99% for another classification and 89% for the validation dataset was achieved [15].

In another study on diabetes prediction, an innovative diabetes prediction model was presented using a set of machine learning techniques such as Logistic Regression, SVM, Naïve Bayes, and Random Forest. In addition to these basic techniques, an impressive accuracy rate of 95.4% was achieved through ensemble learning using CatBoost to further improve prediction accuracy and robustness [18].

In another study examining diabetes using data mining techniques, it was shown that the stacking ensemble technique, especially when combined with hyperparameter tuning, improved the prediction performance of machine learning models [20].

In another study, different methods were compared in terms of their performance regarding diabetes by using particle swarm optimization (PSO) for feature selection. Three medical datasets were used to compare the performance of the methods. According to the findings, Decision Tree, Random Forest, and Naïve Bayes provided the highest accuracy with the lowest error rate [23].

In a similar study, machine learning techniques were discussed and an accuracy value above 95% was obtained using diabetes data. The data collected from Iraqi diabetic patients were subjected to various preprocessing steps before classification, including feature transformation, missing value imputation, data normalization and standardization, feature selection, and k-fold cross-validation [21].

In a study analyzing diabetes datasets to effectively manage diabetes, data mining and machine learning techniques were used. It was stated that patient information is important for diagnosis and prognosis of diabetes in decision-making. Three imbalanced diabetes datasets were used and the datasets were balanced with the synthetic minority oversampling technique. It was stated that balanced datasets provided higher accuracy, recall, precision, and F1 score compared to imbalanced datasets. The Random Forest algorithm was reported to perform well on balanced datasets [26].

In another study involving data mining, it was aimed to explore the complications of diabetes mellitus to find a relationship between gender, age, and occupation factors and diagnostic data. In this study, an algorithmic analysis was conducted to find association rules between the data of diabetic (DM) patients and complications such as renal, ophthalmic, neurological, and peripheral circulation. In other words, the relationships of diabetes with complications like kidney, eye, neurological, and peripheral circulation were examined and co-occurrence effects were interpreted [27].

In another study that examined rare patterns for diabetic complications through data mining (association analysis), a different support value was proposed. It was stated that interesting relationships were discovered between diabetic arthropathy and gastroparesis with neurological symptoms; retinopathy with renal symptoms; ketoacidosis and retinopathy with gastroparesis; and hyperglycemia, peripheral circulation disorder, heart disease and neurological symptoms with skin complications [28].

As seen in the literature, researchers have started to use data related to parameters that may lead to diabetes through data mining techniques. However, it is seen that different parameters were used in these studies, and in some of them, blood test data were also included among the parameters. Blood test data are laborious in terms of time and cost [24]. In addition, in such studies, the effect of each parameter is evaluated individually, while the combined effect of parameters is ignored. In some cases, the high frequency of symptoms alone may not be meaningful, but when combined, they may trigger the disease or pose a risk. Conversely, some symptoms may occur at a low frequency, but when combined with other symptoms, they may trigger the disease.

Based on this need, unlike existing studies, this study evaluates the combined effect of symptoms for the early detection of diabetes. Although association analyses regarding diabetic complications are also present in the literature, these studies mostly examine the co-occurrence of diabetes with the development of other diseases. In contrast, this study investigates the combination of symptoms other than blood tests that directly affect the development of diabetes. An increasing number of sequential associations have also been evaluated.

## Material method

This study uses a dataset of 520 samples collected from Sylhet Diabetes Hospital in Sylhet, Bangladesh. The dataset is based on a real dataset from the UCI Machine Learning Database [29]. The dataset contains 16 variables (symptoms) that can be associated with diabetes. All variables are categorically coded and given a value of zero (0) for the absence of the symptom and one (1) for the presence of the symptom. For age and gender variables, individuals aged 47 years and older and individuals of female gender were coded as one (1). In the ROC analysis of whether diabetes was positive or not, 47.5 was set as the cutoff point for age. The specified cutoff value was calculated based on the current dataset. According to the results of the ROC analysis, the area under the curve (AUC) was found to be 0.6 with a p-value of 0.012. This value corresponds to the highest sensitivity and specificity. There is a statistically significant difference in the mean age between individuals with positive and negative diabetes status. When the mean ages of the two groups are examined, it is observed that the specified cutoff value is supported by the dataset.

The mean age of diabetes-positive individuals was 49.07 ± 12.1 years, while the mean age of diabetes-negative individuals was 46.36 ± 12.1 years, and this difference was found to be statistically significant (*p* < 0.05). In the data set, it was observed that the rate of diabetes was higher in women than in men (*p* < 0.05), and therefore women were considered as the positive group.

First, frequency analysis was performed to examine the distribution of variables, and the relationship of each variable with diabetes was evaluated using chi-square analysis. Then, association analysis was performed to identify symptoms associated with diabetes. For this purpose, the associations between symptoms were analyzed using the apriori algorithm. In the analysis conducted using the R programming language, the necessary algorithm was first provided by installing the “arules” package using the install.packages(“arules”) command, and then loading it into the working environment with the library(arules) command. The diabetes dataset, which forms the basis of the analysis, was imported into the program and converted into a data frame using the as.data.frame function. Subsequently, the as.factor function was applied to define the variables to be used in the analysis as categorical.

To be used in association rule mining, the data were transformed into transactions format via the as(“data”, “transactions”) function. This transformation is necessary for the application of the apriori algorithm.

After data preparation, the apriori function was used to target the positive condition of diabetes; meaningful association rules were extracted by specifying certain support and confidence values. The obtained rules were first sorted using the sort() function based on the defined criteria, and then examined in detail using the inspect() command. Within the scope of this analysis, all symptoms present in the dataset were included in the evaluation, and no missing values were found in the dataset.

**Fig. 1:**
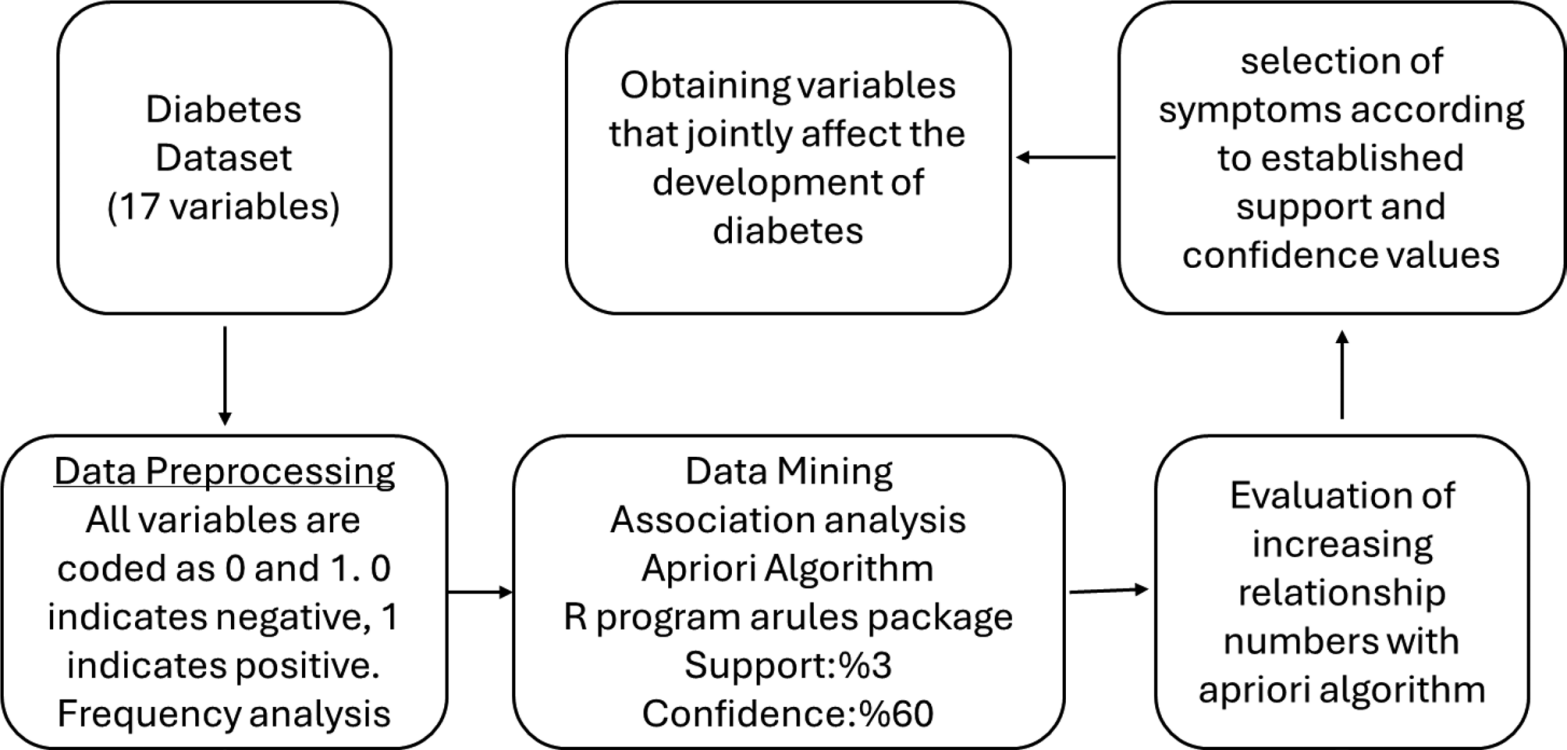
Analysis flow chart

In the algorithm application, the minimum support and confidence values were set as 3 and 60%, respectively, and rules below these values were not considered. Analysis results were interpreted based on the rules with the highest support, confidence and lift values. SPSS 20 (Statistical Package for the Social Sciences) and R 4.3.2 software were used for data analysis. The flow chart prepared for association analysis is shown in Fig. 1.

### Association analysis (apriori algorithm)

The Apriori algorithm is one of the most widely used methods for association rule mining. This algorithm is an association rule algorithm used to identify clusters of items that frequently appear together in large data sets. It is especially preferred in applications such as market basket analysis to identify items that frequently occur together. The Apriori algorithm first identifies groups of frequently occurring items in the dataset and then extracts the rules that determine the relationships between these groups of items [30, 31]. After finding the frequent itemsets, the algorithm generates association rules such as “If A, then B” from these sets. Each association rule is evaluated with metrics such as support, confidence and lift. The Apriori algorithm identifies and analyzes itemsets whose support value is above a certain threshold [32, 33].

Support refers to the rate at which an item set appears in the dataset. This metric indicates the prevalence of a rule in the dataset. A high support value means that the rule is seen more frequently, and this usually results in rules being among the more popular or common items. The support value is calculated by the following Eq. (1) [31].

1 $$Support\left( A \right) = {{Number\,of\,transactions\,A\,has\,gone\,through} \over {Total\,number\,of\,transactions}}$$

For example, if 20 out of 100 patients have both diabetes and obesity, the support value is calculated as: support = 20/100 = 0.2 (20%).

The Apriori algorithm then proceeds to find Frequent Itemsets. The algorithm identifies itemsets that meet the minimum support threshold (min_support). During this process, the most frequent itemsets are identified by evaluating different combinations.

Another important metric is confidence. Confidence is a metric that helps determine the accuracy of a rule and indicates the probability that given a set of items, the other item will also occur. A high confidence value means that when item A (symptom) is present, item B is also likely to be present, indicating that the relationship is strong. The confidence value is calculated by Eq. (2) below: The proportion of co-occurrence of items A and B divided by the proportion of occurrence of item A alone [30].

2 $$Confidence\left( {A \to B} \right) = {{Support\left( {A \cap B} \right)} \over {Support\left( A \right)}}$$

Lift is a measure that assesses the strength of rules compared to random selection. Lift indicates how different the relationship between items (symptoms) is from their co-occurrence by chance alone. Lift is calculated by the following Eq. (3) [34].

3 $$\eqalign{ Lift\left( {A \to B} \right) & = {{Confidence\left( {A \to B} \right)} \over {Support\left( B \right)}} \cr & = {{Support\left( {A \cap B} \right)} \over {Support\left( A \right)xSupport\left( B \right)}} \cr} $$

Lift > 1: In this case, there is a strong association between items and the association is significant. The probability of items A and B (symptoms) being together is higher than the probability of them being independent of each other.

Lift = 1: Items are randomly related and items A and B are independent of each other, i.e. there is no significant relationship between these two items.

Lift < 1: In this case, there may be a negative relationship between the items. There is a negative association between items A and B, meaning that when one item (symptom) occurs, the other is less likely to occur.

To briefly summarize these three metrics: Support indicates the prevalence of the rules; confidence indicates the accuracy of the rules; and lift indicates the strength and significance of the relationship between the two items [30]. In this study, the rules with the highest values for these three metrics were identified and the associations were interpreted.

Working Steps of the Algorithm:

- Determination of minimum support and minimum confidence values.
- Calculating the support values of each item in the item sets.
- Disabling items with support values lower than the minimum support threshold.
- Creating binary associations by considering single associations.
- Removing the itemsets with support values lower than the minimum support threshold.
- Creation of ternary associations.
- Exclusion of ternary associations that do not exceed the minimum support value.
- Extraction of association rules from triple associations.

The algorithm ranks the results by determining the most meaningful rules [31].

The Apriori algorithm has a simple and straightforward structure, and this simplicity facilitates the implementation of the algorithm and the interpretation of the results obtained. Especially for small and medium-sized datasets, the Apriori algorithm provides effective and reliable results. In large-scale datasets, it provides a high accuracy rate. However, the processing time may be longer for large datasets because the algorithm’s running speed may decrease as the number of combinations increases. Also, if the minimum support value is not set well, there may be too many or too few rules. The Apriori algorithm is a powerful method in data mining, allowing to generate meaningful association rules by analyzing frequent itemsets.

## Results

**Fig. 2:**
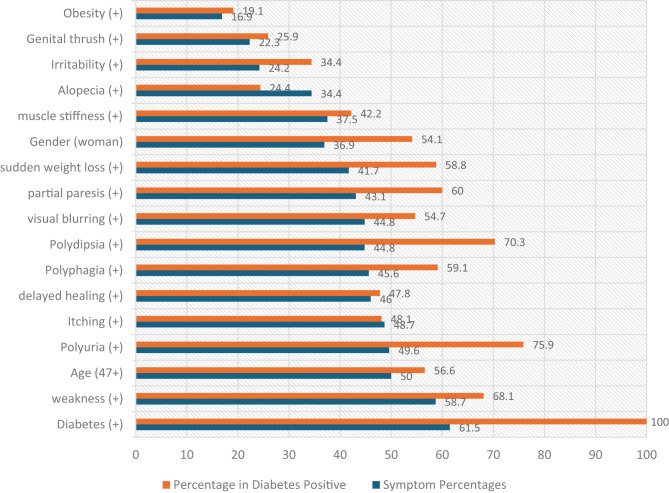
Scatter graph of symptoms

In this study, we tried to determine the symptoms that affect diabetes positivity together. In this context, variables that may be associated with diabetes were examined. The dataset consists of 17 variables, the sample size is 520 and there is no missing data in the dataset. The variables of the real dataset obtained from the UCI Machine Learning Database and the descriptive statistics of these variables are presented in Table 1 and Fig. 2. Symptoms are ranked according to their positivity rates. The percentages in parentheses in Table 1 show the positive rates of other symptoms among individuals with positive diabetes. The rate of individuals positive for diabetes is 61.5%. “Weakness was the most common symptom (58.7%), while excessive urination (polyuria) was the most common symptom (75.9%) in individuals with positive diabetes. Excessive urination was observed in approximately 50% of patients overall. Excessive drinking of water (polydipsia) was the seventh most common symptom (44.8%) and the second most common symptom in diabetes-positive individuals (70.3%). The top five symptoms with the highest rates in individuals with positive diabetes were excessive urination (75.9%), excessive drinking (70.3%), weakness (68.1%), partial paresis (60%) and overeating (polyphagia) (59.1%). Although partial paresis was the ninth most common symptom overall, it was observed in 60% of individuals with positive diabetes. Among the other symptoms, obesity (16.9%), genital thrush (22.3%) and irritability (24.2%) had the lowest prevalence rates.

**Table 1:** Descriptive statistics of variables

Variables	Frequency	Percent (*)
Diabetes (+)	320	61.5 (100)
weakness (+)	305	58.7 (**68.1**)
Age (47+)	260	50.0 (56.6)
Polyuria (+)	258	49.6 (**75.9**)
Itching (+)	253	48.7 (48.1)
delayed healing (+)	239	46.0 (47.8)
Polyphagia (+)	237	45.6 (**59.1**)
Polydipsia (+)	233	44.8 (**70.3**)
visual blurring (+)	233	44.8 (54.7)
partial paresis (+)	224	43.1 (**60.0**)
sudden weight loss (+)	217	41.7 (58.8)
Gender (woman)	192	36.9 (54.1)
muscle stiffness (+)	195	37.5 (42.2)
Alopecia (+)	179	34.4 (24.4)
Irritability (+)	126	24.2 (34.4)
Genital thrush (+)	116	22.3 (25.9)
Obesity (+)	88	16.9 (19.1)

* Percentage of other symptoms in those with diabetes positive

The relationships between diabetes and other symptoms were analyzed using chi-square analysis and the results are presented in Table 2. When the relationship between symptoms and diabetes was evaluated, no statistically significant relationship was found between itching, delayed healing and obesity symptoms (*p* > 0.05). A significant association was found between all other symptoms and diabetes (*p* < 0.05). Gender-based analysis showed that 90.1% of women were diabetes positive and 55.2% of men were diabetes negative.

**Table 2:** Association between diabetes variable and other symptoms

			Diabetes		Total	p
			-	+		
Gender	woman	Count	19	173	192	< 0.01
		% within Gender	9.9%	90.1%	100.0%	
		% within class	9.5%	54.1%	36.9%	
	man	Count	181	147	328	
		% within Gender	55.2%	44.8%	100.0%	
		% within class	90.5%	45.9%	63.1%	
Polyuria	-	Count	185	77	262	< 0.01
		% within Polyuria	70.6%	29.4%	100.0%	
		% within class	92.5%	24.1%	50.4%	
	+	Count	15	243	258	
		% within Polyuria	5.8%	94.2%	100.0%	
		% within class	7.5%	75.9%	49.6%	
Polydipsia	-	Count	192	95	287	< 0.01
		% within Polydipsia	66.9%	33.1%	100.0%	
		% within class	96.0%	29.7%	55.2%	
	+	Count	8	225	233	
		% within Polydipsia	3.4%	96.6%	100.0%	
		% within class	4.0%	70.3%	44.8%	
sudden weight loss	-	Count	171	132	303	< 0.01
		% within sudden weight loss	56.4%	43.6%	100.0%	
		% within class	85.5%	41.3%	58.3%	
	+	Count	29	188	217	
		% within sudden weight loss	13.4%	86.6%	100.0%	
		% within class	14.5%	58.8%	41.7%	
weakness	-	Count	113	102	215	< 0.01
		% within weakness	52.6%	47.4%	100.0%	
		% within class	56.5%	31.9%	41.3%	
	+	Count	87	218	305	
		% within weakness	28.5%	71.5%	100.0%	
		% within class	43.5%	68.1%	58.7%	
Polyphagia	-	Count	152	131	283	< 0.01
		% within Polyphagia	53.7%	46.3%	100.0%	
		% within class	76.0%	40.9%	54.4%	
	+	Count	48	189	237	
		% within Polyphagia	20.3%	79.7%	100.0%	
		% within class	24.0%	59.1%	45.6%	
Genital thrush	-	Count	167	237	404	0.012
		% within Genital thrush	41.3%	58.7%	100.0%	
		% within class	83.5%	74.1%	77.7%	
	+	Count	33	83	116	
		% within Genital thrush	28.4%	71.6%	100.0%	
		% within class	16.5%	25.9%	22.3%	
visual blurring	-	Count	142	145	287	< 0.01
		% within visual blurring	49.5%	50.5%	100.0%	
		% within class	71.0%	45.3%	55.2%	
	+	Count	58	175	233	
		% within visual blurring	24.9%	75.1%	100.0%	
		% within class	29.0%	54.7%	44.8%	
Itching	-	Count	101	166	267	0.760
		% within Itching	37.8%	62.2%	100.0%	
		% within class	50.5%	51.9%	51.3%	
	+	Count	99	154	253	
		% within Itching	39.1%	60.9%	100.0%	
		% within class	49.5%	48.1%	48.7%	
Irritability	-	Count	184	210	394	< 0.01
		% within Irritability	46.7%	53.3%	100.0%	
		% within class	92.0%	65.6%	75.8%	
	+	Count	16	110	126	
		% within Irritability	12.7%	87.3%	100.0%	
		% within class	8.0%	34.4%	24.2%	
delayed healing	-	Count	114	167	281	0.284
		% within delayed healing	40.6%	59.4%	100.0%	
		% within class	57.0%	52.2%	54.0%	
	+	Count	86	153	239	
		% within delayed healing	36.0%	64.0%	100.0%	
		% within class	43.0%	47.8%	46.0%	
partial paresis	-	Count	168	128	296	< 0.01
		% within partial paresis	56.8%	43.2%	100.0%	
		% within class	84.0%	40.0%	56.9%	
	+	Count	32	192	224	
		% within partial paresis	14.3%	85.7%	100.0%	
		% within class	16.0%	60.0%	43.1%	
muscle stiffness	-	Count	140	185	325	0.005
		% within muscle stiffness	43.1%	56.9%	100.0%	
		% within class	70.0%	57.8%	62.5%	
	+	Count	60	135	195	
		% within muscle stiffness	30.8%	69.2%	100.0%	
		% within class	30.0%	42.2%	37.5%	
Alopecia	-	Count	99	242	341	< 0.01
		% within Alopecia	29.0%	71.0%	100.0%	
		% within class	49.5%	75.6%	65.6%	
	+	Count	101	78	179	
		% within Alopecia	56.4%	43.6%	100.0%	
		% within class	50.5%	24.4%	34.4%	
Obesity	-	Count	173	259	432	0.1
		% within Obesity	40.0%	60.0%	100.0%	
		% within class	86.5%	80.9%	83.1%	
	+	Count	27	61	88	
		% within Obesity	30.7%	69.3%	100.0%	
		% within class	13.5%	19.1%	16.9%	
Age	16–47	Count	121	139	260	< 0.01
		% within yas47	46.5%	53.5%	100.0%	
		% within class	60.5%	43.4%	50.0%	
	47+	Count	79	181	260	
		% within yas47	30.4%	69.6%	100.0%	
		% within class	39.5%	56.6%	50.0%	

While 94.2% of individuals with positive polyuria symptoms were diabetes positive, 70.6% of those with negative polyuria symptoms were diabetes negative. While 96.6% of those with positive polydipsia symptoms were positive for diabetes, 66.9% of those with negative symptoms were negative for diabetes. Among those with a positive Sudden weight loss symptom, 86.6% were positive for diabetes, while 56.4% of those with a negative symptom were negative for diabetes. While 71.5% of the individuals with positive Weakness symptom had positive diabetes, 52.6% of the negative ones had negative diabetes. While 79.7% of those with positive polyphagia had positive diabetes, 53.7% of those with negative polyphagia had negative diabetes.

Among those with positive genital thrush symptoms, 71.6% had positive diabetes, while 41.3% of those with negative symptoms had negative diabetes. Of those with positive visual blurring symptoms, 75.1% had positive diabetes, while 49.5% of those with negative symptoms had negative diabetes. While 87.3% of the individuals with positive Irritability symptom had positive diabetes, 46.7% of the negative ones had negative diabetes. Among those with positive partial paresis symptom, 85.7% had positive diabetes, while 56.8% of those with negative symptoms had negative diabetes. Of those with positive muscle stiffness symptom, 69.2% had positive diabetes, while 43.1% of those with negative symptom had negative diabetes.

Alopecia symptom showed an inverse relationship with diabetes. Diabetes was found to be negative in 56.4% of individuals with positive alopecia and positive in 71% of those with negative alopecia. These results suggest that the alopecia symptom has an inverse relationship with diabetes; that is, the rate of diabetes negativity was higher when alopecia was positive and the rate of diabetes positivity was higher when alopecia was negative.

According to the age group-based assessment, 69.6% of individuals aged 47 years and over had positive diabetes, while 46.5% of individuals aged 16–47 years had negative diabetes.

The results of the Apriori algorithm are presented in Tables 3, 4, 5, 6, 7 and 8. Association rules were analyzed in order of association, with binary symptoms associated with diabetes first, followed by triadic and ascending associations. The algorithm was applied with a minimum of 3% support and 60% confidence and the association rules with the highest support, confidence and lift values were ranked. Table 3 shows the association values of other symptoms with diabetes alone. The LHS (left hand side) and RHS (right hand side) columns in this table refer to the symptoms in the rules. Positive symptoms in the LHS column are shown in association with the item in the RHS column. Alopecia symptom was not included in the rules because its support and confidence values for co-occurrence with diabetes were below the minimum limits.

**Table 3:** Binary association analysis results

LHS	RHS	supp	conf	lift
{Polyuria = 1}	{Diabetes = 1}	0.47	0.94	1.53
{Polydipsia = 1}	{Diabetes = 1}	0.43	0.97	1.57
{weakness = 1}	{Diabetes = 1}	0.42	0.72	1.16
{partial.paresis = 1}	{Diabetes = 1}	0.37	0.86	1.39
{Polyphagia = 1}	{Diabetes = 1}	0.36	0.80	1.30
{sudden.weight.loss = 1}	{Diabetes = 1}	0.36	0.87	1.41
{Age = 1}	{Diabetes = 1}	0.35	0.70	1.13
{Gender = 1}	{Diabetes = 1}	0.33	0.90	1.46
{Itching = 1}	{Diabetes = 1}	0.30	0.61	0.99
{visual.blurring = 1}	{Diabetes = 1}	0.34	0.75	1.22
{delayed.healing = 1}	{Diabetes = 1}	0.29	0.64	1.04
{muscle.stiffness = 1}	{Diabetes = 1}	0.26	0.69	1.13
{Irritability = 1}	{Diabetes = 1}	0.21	0.87	1.42
{Genital.thrush = 1}	{Diabetes = 1}	0.16	0.72	1.16
{Obesity = 1}	{Diabetes = 1}	0.12	0.69	1.12

When binary associations were analyzed, it was observed that the most important symptoms were polyuria and polydipsia. In the dataset, the co-positive rate of polyuria and diabetes was 47% and the co-positive rate of polydipsia was 43%. Looking at the confidence values, the presence of strong associations between both symptoms became evident. Individuals with positive polyuria had a 94% probability of having diabetes, while individuals with positive polydipsia had a 97% probability of having diabetes. Looking at the lift values, it was found that individuals with polyuria symptoms were 1.53 times more likely to have diabetes than a randomly selected individual, while individuals with polydipsia symptoms were 1.57 times more likely to have diabetes. Both symptoms are strongly associated with diabetes. The confidence value for the polydipsia symptom in particular was as high as 97%, indicating that the diagnosis of diabetes is almost certain when this symptom is present. Lift values greater than 1 indicate that both symptoms are factors that increase the risk of diabetes.

When the relationship between itching symptom and diabetes was analyzed, no significant relationship was found in terms of lift value. However, when the rates of the variables alone are considered, it is observed that the positive rate of itching symptom is higher than the positive rate of polydipsia symptom. This clearly demonstrates the importance of evaluating the association effect.

The results of triple associations are presented in Table 4. The positive rate of polyuria, polydipsia and diabetes together was 37% in the dataset. The confidence value of this triple association indicates that every individual with polyuria and polydipsia symptoms has diabetes. In other words, every individual with these two symptoms is considered to have a definite diagnosis of diabetes in the dataset. The Lift value reveals that compared to a random individual, individuals with polyuria and polydipsia symptoms are 1.63 times more likely to have diabetes. Furthermore, it was observed that all of the women who were positive for polyuria had diabetes. While the proportion of female individuals with positive polyuria and diabetes was 25%, it was determined that a female individual with positive polyuria symptoms was 1.63 times more likely to have diabetes.

**Table 4:** Triple association analysis results

LHS	RHS	supp	conf	lift
{Polyuria = 1, Polydipsia = 1}	{Diabetes = 1}	0.37	1.00	1.63
{Polyuria = 1, weakness = 1}	{Diabetes = 1}	0.34	0.96	1.56
{Polydipsia = 1, weakness = 1}	{Diabetes = 1}	0.34	0.98	1.59
{Polyuria = 1, sudden.weight.loss = 1}	{Diabetes = 1}	0.31	0.98	1.59
{Polyuria = 1, partial.paresis = 1}	{Diabetes = 1}	0.31	0.96	1.56
{Polyuria = 1, Polyphagia = 1}	{Diabetes = 1}	0.30	0.94	1.53
{Polydipsia = 1, partial.paresis = 1}	{Diabetes = 1}	0.29	0.97	1.58
{sudden.weight.loss = 1, weakness = 1}	{Diabetes = 1}	0.28	0.91	1.48
{Age = 1, Polyuria = 1}	{Diabetes = 1}	0.28	0.93	1.51
{weakness = 1, partial.paresis = 1}	{Diabetes = 1}	0.28	0.89	1.44
{Polydipsia = 1, sudden.weight.loss = 1}	{Diabetes = 1}	0.28	0.98	1.59
{Polydipsia = 1, Polyphagia = 1}	{Diabetes = 1}	0.28	0.99	1.61
{Polydipsia = 1, visual.blurring = 1}	{Diabetes = 1}	0.27	0.95	1.55
{Age = 1, Polydipsia = 1}	{Diabetes = 1}	0.27	0.97	1.57
{weakness = 1, Polyphagia = 1}	{Diabetes = 1}	0.27	0.86	1.39
{weakness = 1,visual.blurring = 1}	{Diabetes = 1}	0.26	0.78	1.26
{Polyuria = 1,visual.blurring = 1}	{Diabetes = 1}	0.26	0.92	1.50
{Polyphagia = 1, partial.paresis = 1}	{Diabetes = 1}	0.26	0.89	1.45
{Age = 1, weakness = 1}	{Diabetes = 1}	0.25	0.72	1.17
{Gender = 1, Polyuria = 1}	{Diabetes = 1}	0.25	1.00	1.63
{visual.blurring = 1, partial.paresis = 1}	{Diabetes = 1}	0.25	0.87	1.42

The combined positivity rate of the triple symptoms of polyuria-weakness-diabetes and polydipsia-weakness-diabetes was 34%. An individual with positive polydipsia and weakness symptoms was 1.59 times more likely to have diabetes. The rules with the highest support, confidence and lift values for the triple association are presented in detail in Table 4. The impact of other symptoms can also be examined through this table. In particular, symptoms such as Polyuria, Polydipsia and weakness stand out as factors that exhibit a strong association with the diagnosis of diabetes. Other symptoms have lower confidence and lift values and may be relatively less effective in predicting diabetes.

The results of the analysis of the quartet associations presented in Table 5 provide a deeper insight into the relationship between diabetes and symptoms. In this analysis, the rule with the highest support, confidence and lift values is the association between Polyuria, Polydipsia, Weakness and Diabetes. The co-occurrence rate of these four symptoms was calculated to be 30% in the dataset. In particular, all individuals with Polyuria, Polydipsia and Weakness symptoms were positively diagnosed with diabetes. Similarly, all individuals with Polyuria, Polydipsia and Partial Paresis symptoms also had a positive diagnosis of diabetes. In both rules, the co-occurrence of these symptoms increases the likelihood of diabetes by 1.63 times.

**Table 5:** Quartet association analysis results

LHS	RHS	supp	conf	lift
{Polyuria = 1,Polydipsia = 1, weakness = 1}	{Diabetes = 1}	0.30	1.00	1.63
{Polyuria = 1,Polydipsia = 1,partial.paresis = 1}	{Diabetes = 1}	0.27	1.00	1.63
{Polydipsia = 1, weakness = 1,partial.paresis = 1}	{Diabetes = 1}	0.25	0.98	1.59
{Polyuria = 1,Polydipsia = 1,sudden.weight.loss = 1}	{Diabetes = 1}	0.25	1.00	1.63
{Polyuria = 1,weakness = 1,partial.paresis = 1}	{Diabetes = 1}	0.25	1.00	1.63
{Polyuria = 1,Polydipsia = 1,Polyphagia = 1}	{Diabetes = 1}	0.25	1.00	1.63
{Polyuria = 1,sudden.weight.loss = 1,weakness = 1}	{Diabetes = 1}	0.24	0.97	1.58
{Polyuria = 1,Polydipsia = 1,visual.blurring = 1}	{Diabetes = 1}	0.23	1.00	1.63
{Polydipsia = 1,sudden.weight.loss = 1,weakness = 1}	{Diabetes = 1}	0.23	0.98	1.59
{Polydipsia = 1,weakness = 1,visual.blurring = 1}	{Diabetes = 1}	0.23	0.97	1.57
{Polydipsia = 1,Polyphagia = 1,partial.paresis = 1}	{Diabetes = 1}	0.23	1.00	1.63
{Age = 1,Polyuria = 1,Polydipsia = 1}	{Diabetes = 1}	0.23	1.00	1.63

Furthermore, the Partial Paresis symptom, when considered alone, was 43.1% of the dataset, whereas when considered in combination with Polyuria and Polydipsia symptoms, a 100% confidence value was obtained with diabetes. This suggests that the co-occurrence of these three symptoms strongly indicates diabetes and that this combination of symptoms is an important predictor for the diagnosis of diabetes. These findings suggest that symptom combinations in particular provide a stronger and more reliable indicator of the presence of diabetes. These combinations are important tools in clinical diagnostic processes.

The analysis of the five-fold associations presented in Table 6 reveals the effect of diabetes-related symptom combinations in more detail. The rule with the highest values consists of the combination of Polyuria, Polydipsia, Weakness, Partial Paresis and Diabetes symptoms. The rate of positive occurrence of this five-fold symptom combination in the data set was determined as 23%. In addition, all individuals with the symptoms of Polyuria, Polydipsia, Weakness and Partial Paresis were diagnosed as positive for diabetes, which was confirmed with a confidence value of 100%. The combination of these four symptoms increases the probability of being positive for diabetes by 1.63 times.

**Table 6:** Five-way association analysis results

LHS	RHS	supp	conf	lift
{Polyuria = 1,Polydipsia = 1,weakness = 1,partial.paresis = 1}	{Diabetes = 1}	0.23	1.00	1.63
{Polyuria = 1,Polydipsia = 1,sudden.weight.loss = 1,weakness = 1}	{Diabetes = 1}	0.21	1.00	1.63
{Polyuria = 1,Polydipsia = 1,Polyphagia = 1,partial.paresis = 1}	{Diabetes = 1}	0.21	1.00	1.63
{Polyuria = 1,Polydipsia = 1,weakness = 1,visual.blurring = 1}	{Diabetes = 1}	0.21	1.00	1.63
{Polyuria = 1,Polydipsia = 1,sudden.weight.loss = 1,partial.paresis = 1}	{Diabetes = 1}	0.20	1.00	1.63
{Polyuria = 1,Polydipsia = 1,weakness = 1,Polyphagia = 1}	{Diabetes = 1}	0.20	1.00	1.63
{Polydipsia = 1, weakness = 1,Polyphagia = 1,partial.paresis = 1}	{Diabetes = 1}	0.19	1.00	1.63
{Gender = 1,Polyuria = 1,Polydipsia = 1,partial.paresis = 1}	{Diabetes = 1}	0.19	1.00	1.63
{Polyuria = 1,sudden.weight.loss = 1,weakness = 1,partial.paresis = 1}	{Diabetes = 1}	0.19	1.00	1.63
{Polydipsia = 1,sudden.weight.loss = 1,weakness = 1,partial.paresis = 1}	{Diabetes = 1}	0.19	0.97	1.58
{Polyuria = 1,Polydipsia = 1,sudden.weight.loss = 1,Polyphagia = 1}	{Diabetes = 1}	0.19	1.00	1.63
{Age = 1,Polyuria = 1,Polydipsia = 1,weakness = 1}	{Diabetes = 1}	0.19	1.00	1.63

However, the incidence rate of the Sudden Weight Loss symptom is given as 41.7% in Table 1, which has a lower prevalence compared to other symptoms. However, when seen together with the symptoms of Polyuria, Polydipsia and Weakness, the diagnosis of diabetes was determined with 100% accuracy. This five-fold association significantly increases the probability of being positive for diabetes by 1.63 times. These findings obtained with association analysis show that the co-occurrence of certain symptoms can significantly increase the accuracy of diabetes diagnosis and that symptom combinations can be used in clinical practices in diagnostic processes.

The analysis of the associations evaluated with increasing symptom count shows that the rate of co-occurrence of the rule tends to decrease in the data set, but the confidence and lift values increase significantly. The analysis results of the 6- and seven-fold associations are presented in Table 7. In the six-fold association rule showing the highest values, the rate of co-occurrence of Polyuria, Polydipsia, Sudden Weight Loss, Weakness, Partial Paresis and Diabetes symptoms was determined to be 17%. Regarding this rule, it was observed that all individuals in whom the symptoms in the LHS (left-hand side) column were seen together were positive for diabetes and that these symptoms increased the probability of diabetes by 1.63 times.

**Table 7:** Six and seven-fold association analysis results

LHS	RHS	supp	conf	lift
{Polyuria = 1, Polydipsia = 1, sudden.weight.loss = 1,weakness = 1,partial.paresis = 1}	{Diabetes = 1}	0.17	1.00	1.63
{Polyuria = 1, Polydipsia = 1,weakness = 1,Polyphagia = 1,partial.paresis = 1}	{Diabetes = 1}	0.17	1.00	1.63
{Polyuria = 1,Polydipsia = 1,weakness = 1, visual.blurring = 1,partial.paresis = 1}	{Diabetes = 1}	0.16	1.00	1.63
{Age = 1,Polyuria = 1,Polydipsia = 1,weakness = 1,partial.paresis = 1}	{Diabetes = 1}	0.15	1.00	1.63
{Gender = 1,Polyuria = 1,Polydipsia = 1,Polyphagia = 1,partial.paresis = 1}	{Diabetes = 1}	0.15	1.00	1.63
{Gender = 1,Polyuria = 1, Polydipsia = 1,sudden.weight.loss = 1,partial.paresis = 1}	{Diabetes = 1}	0.15	1.00	1.63
{Polyuria = 1,Polydipsia = 1,sudden.weight.loss = 1,weakness = 1,Polyphagia = 1,partial.paresis = 1}	{Diabetes = 1}	0.13	1.00	1.63
{Gender = 1,Polyuria = 1,Polydipsia = 1,weakness = 1,Polyphagia = 1,partial.paresis = 1}	{Diabetes = 1}	0.12	1.00	1.63
{Polyuria = 1,Polydipsia = 1,sudden.weight.loss = 1,weakness = 1,visual.blurring = 1,partial.paresis = 1}	{Diabetes = 1}	0.12	1.00	1.63
{Gender = 1,Polyuria = 1,Polydipsia = 1,sudden.weight.loss = 1,Polyphagia = 1,partial.paresis = 1}	{Diabetes = 1}	0.12	1.00	1.63
{Gender = 1,Polyuria = 1,Polydipsia = 1,sudden.weight.loss = 1,weakness = 1,partial.paresis = 1}	{Diabetes = 1}	0.12	1.00	1.63
{Gender = 1,Polyuria = 1,Polydipsia = 1,weakness = 1, visual.blurring = 1,partial.paresis = 1}	{Diabetes = 1}	0.11	1.00	1.63

The rule with the highest support value for the seven-fold association is the rule in which the symptoms of Polyuria, Polydipsia, Sudden Weight Loss, Weakness, Polyphagia, Partial Paresis and Diabetes are seen together. The positive co-occurrence rate of this rule is 13%, the confidence value is 100% and the lift value is 1.63. This seven-fold association rule, unlike the previous six-fold rule, was formed by adding the Polyphagia symptom. Interestingly, while the lift value of the Polyphagia symptom in its dual association with diabetes was 1.30, the lift value increased to 1.63 with the inclusion of this symptom in the seven-fold association. This situation shows that the effect of the Polyphagia symptom on the diagnosis of diabetes increases and the interaction between the symptoms is strengthened. The findings reveal that the role of individual symptoms and their combinations in the diagnosis of diabetes differs. Association analysis can contribute to clinical decision support systems, especially in the early diagnosis of multi-symptom diseases such as diabetes.

**Fig. 3:**
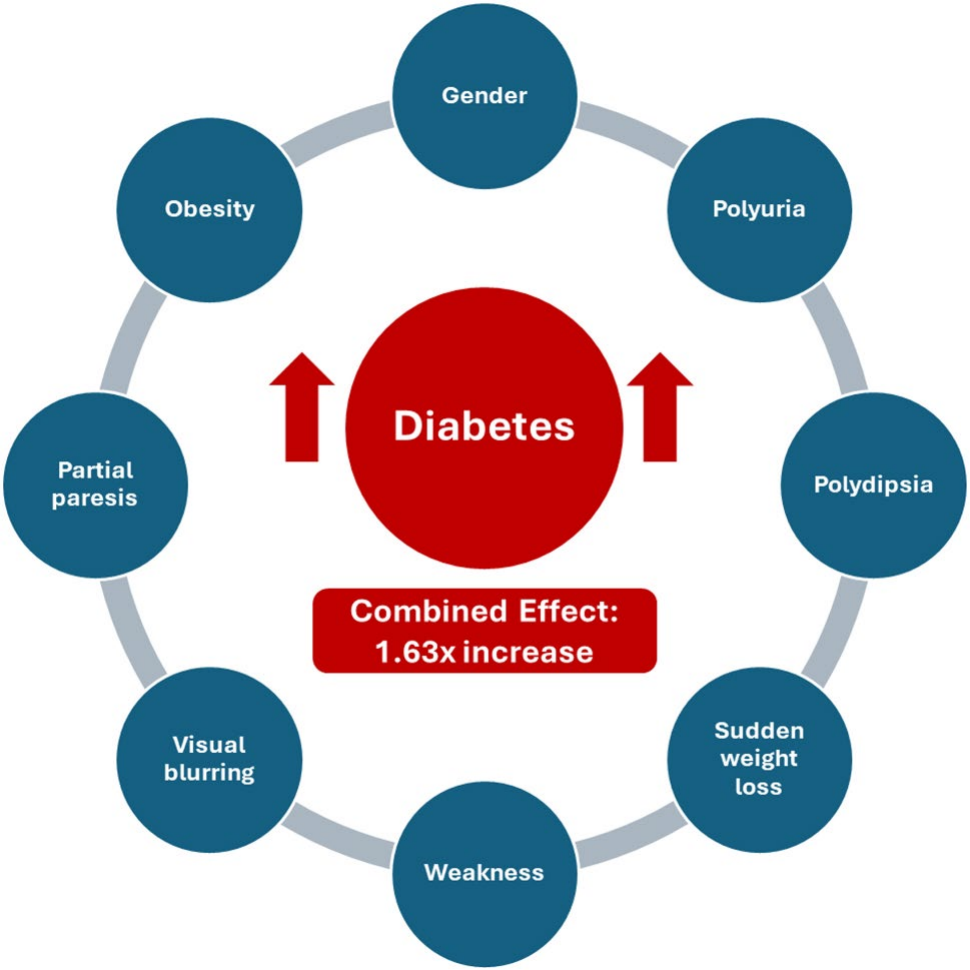
Symptoms affecting the development of diabetes

Finally, the results obtained by examining the eight and nine associations are presented in Table 8 and Fig. 3. In this analysis, only significant associations were focused on, considering the minimum support (3%) and confidence (60%) values. While the highest value rule for the eight association was Gender-Polyuria-Polydipsia-Sudden Weight Loss-Weakness-Visual Blurring-Obesity-Diabetes, the rule for the nine association was Gender-Polyuria-Polydipsia-Sudden Weight Loss-Weakness-Visual Blurring-Partial Paresis-Obesity-Diabetes. These rules show that there is a strong association between the symptoms associated with diabetes. The analysis evaluated the effect of the co-occurrence of symptoms on the diagnosis of diabetes and presented results based on the association metrics obtained thanks to the a priori algorithm. In this regard, the symptoms that should be examined together in the diagnosis of diabetes were determined as Gender, Polyuria, Polydipsia, Sudden Weight Loss, Weakness, Visual Blurring, Partial Paresis and Obesity.

**Table 8:** Results of eight and nine-fold association analysis

LHS	RHS	supp	conf	lift
{Gender = 1,Polyuria = 1,Polydipsia = 1,sudden.weight.loss = 1,weakness = 1,visual.blurring = 1,Obesity = 1}	{Diabetes = 1}	0.04	1.00	1.63
{Gender = 1,Polyuria = 1, sudden.weight.loss = 1,weakness = 1,visual.blurring = 1,partial.paresis = 1,Obesity = 1}	{Diabetes = 1}	0.04	1.00	1.63
{Gender = 1,Polyuria = 1,Polydipsia = 1,sudden.weight.loss = 1,weakness = 1,partial.paresis = 1,Obesity = 1}	{Diabetes = 1}	0.03	1.00	1.63
{Gender = 1, Polyuria = 1,Polydipsia = 1,sudden.weight.loss = 1,visual.blurring = 1,partial.paresis = 1,Obesity = 1}	{Diabetes = 1}	0.03	1.00	1.63
{Gender = 1,Polydipsia = 1,sudden.weight.loss = 1,weakness = 1,visual.blurring = 1,partial.paresis = 1,Obesity = 1}	{Diabetes = 1}	0.03	1.00	1.63
{Gender = 1,Polyuria = 1,Polydipsia = 1,sudden.weight.loss = 1,weakness = 1,visual.blurring = 1,partial.paresis = 1,Obesity = 1}	{Diabetes = 1}	0.03	1.00	1.63

In particular, while the Obesity variable was not found to have a significant relationship with diabetes alone (*p* = 0.1), it was observed that it made a significant contribution to the positive development of diabetes when evaluated together with other symptoms. This finding reveals that the Obesity symptom is an important determinant that strengthens the diagnosis of diabetes when evaluated together with other risk factors.

Association analysis and the findings emphasize the importance of evaluating symptoms together in the diagnosis of diabetes. In addition, determining the effect of combinations of symptoms on the diagnosis of diabetes can contribute to more accurate and early diagnosis processes in clinical practices.

## Discussion

The main purpose of this study is to determine the determinant symptoms for early diagnosis of diabetes, and the association analysis method was used for this purpose. The symptoms that affect the development of diabetes together were analyzed and the role of the co-occurrence of these symptoms in the diagnosis of the disease was evaluated. In this context, the study was built on real patient data obtained from the Sylhet Diabetes Hospital in the city of Sylhet, Bangladesh. The data include the symptoms observed in the patients rather than blood test results. In this way, by helping to diagnose diabetes early through co-occurring symptoms without blood values, fast and less costly results can be obtained and the treatment process can be accelerated. Complications that may develop due to diabetes can be prevented. Considering that diabetes also significantly affects the quality of life in addition to complications and its negative consequences on professional productivity, mental health and family life [1, 35], its early diagnosis is quite important.

Since diabetes has become one of the most common diseases in middle- and low-income countries, medical professionals are looking for reliable methods to predict the diagnosis [23]. There are many studies in the literature on the prevention of diabetes, lifestyle recommendations at a young age, and early diagnosis [36]. In some studies, models have been developed for accurate and early diagnosis of diabetes with different variables using machine learning methods [4, 16, 18, 20, 22]. In some studies, it has been tried to select diabetes-related characteristics (risk factors) through machine learning-based systems [4, 23, 37–39]. However, the difficulties in determining effective biomarkers for the progression of the disease are emphasized [40–42].

This study, unlike others, uses association analysis to determine symptoms associated with diabetes. Because a symptom that is not found significant alone can play an important role in the development of the disease when seen together with other symptoms. A symptom with a low incidence or a symptom that is not significantly related to diabetes can significantly increase the development of the disease when combined with other symptoms. Therefore, it is very important to evaluate the related symptoms together. Age, obesity, weakness, sudden weight loss, polyuria, thirst, constant hunger, vision changes and fatigue are among the potential symptoms of diabetes [4, 10, 11, 21]. Individuals at risk of diabetes should follow these symptoms and have periodic medical check-ups, which may reduce the risks that may arise if the disease is not diagnosed [10]. Similar symptoms were evaluated using the apriori algorithm in this study.

As a result of the association analysis, eight symptoms that affect diabetes together were determined (Table 8). These were found to be gender, polyuria, polydipsia, sudden weight loss, weakness, visual blurring, partial paresis and obesity (Fig. 3). The positive appearance of these eight symptoms together increases the probability of developing diabetes by 1.63 times. Another study that classified diabetes using the same data set with machine learning methods determined gender, polyuria, polydipsia, sudden weight loss, partial paresis, age, alopecia, irritability and polyphagi variables as important features affecting diabetes as a result of feature selection [24]. When two studies using the same data set were examined, while weakness, visual blurring and obesity variables were selected differently in our study, age, alopecia, irritability and polyphagi variables were selected differently in the other study. The variables that were determined to be important in both studies were gender, polyuria, polydipsia, sudden weight loss and partial paresis. The three most important risk factors in the machine learning study were found to be polydipsia, polyuria and gender. In the association analysis, the rule with the highest support, confidence, and lift values was found to be polyuria-polydipsia-weakness in the examination of three symptoms associated with diabetes (Table 5). Based on this, it was concluded that different methods determined different variables as important features. As a result of the chi-square analysis, the relationship between the symptoms of delay healing, itching, and obesity and diabetes was not found to be statistically significant as in the other study (*p* > 0.05). However, while obesity was not found to be significant in the machine learning model, it was seen to have a significant effect on the development of the disease when examined together with other symptoms in the association analysis. This finding reveals the importance of the apriori algorithm working based on the co-occurrence of symptoms rather than individual symptoms.

It was determined that the average age of individuals with positive diabetes diagnosis was statistically significantly higher than those with negative diabetes (*p* < 0.05). While the age variable was determined as an important risk factor in the machine learning study, the lift value of its binary relationship with diabetes was found to be close to 1 in the association analysis. A lift value of 1 indicates that the relationship between the variables is not significant. Since the alopecia variable remained below the minimum threshold values determined in the association analysis, it was not included among the rules evaluated within the scope of the analysis (Table 1). Compared to other symptoms, its incidence in individuals with positive diabetes remained at a low level. In addition, while the rate of diabetes being negative was found to be higher in cases where the alopecia variable was positive, the rate of diabetes being positive was observed to be higher in individuals with negative alopecia. However, it was observed that it was selected among the important risk factors in the machine learning study. It was determined that the polyphagia symptom was observed at a high rate among individuals with positive diabetes diagnosis, and the lift value in its binary relationship with diabetes was 1.30 (Table 1). However, in the analysis where the effect of eight symptoms on diabetes was evaluated together, this symptom was not among the significant variables. Similarly, it was determined that the irritability symptom had a low incidence compared to other variables (Table 1), but it was determined that it was selected as a risk factor in the machine learning study [24].

In another study utilizing a similar dataset, variables related to diabetes diagnosis were evaluated based on statistical significance, with each variable examined independently. As a result, the study recommended the use of 13 symptoms for the diagnosis of diabetes. In comparison, our findings suggest that fewer symptoms may be sufficient in the diagnostic process [43].

In a different study employing data mining techniques, the aim was to explore the relationship between demographic factors such as gender, age, and occupation, and diagnostic data by investigating diabetes-related complications. This study analyzed data from diabetic patients to examine associations between complications related to the kidneys, eyes, neurological system, and peripheral circulation [27]. Another study using association analysis focused on identifying rare patterns among diabetic complications. Notable associations were identified between diabetic arthropathy and gastroparesis with neurological symptoms; retinopathy with kidney-related symptoms; ketoacidosis and retinopathy with gastroparesis; and hyperglycemia, peripheral circulatory disorders, cardiovascular disease, and neurological symptoms with dermatological complications [28]. These studies predominantly focus on the comorbidity of diabetes with other complications. In contrast, our study investigates the combined effect of non-laboratory symptoms on the development of diabetes, aiming to identify symptom combinations that directly influence disease onset.

As a result, instead of costly blood test results, evaluations based on observation of diabetes-related symptoms together show that positive observation of eight symptoms together has a significant effect on the development of diabetes. These symptoms were determined as gender, polyuria, polydipsia, sudden weight loss, weakness, visual blurring, partial paresis and obesity. It is recommended that physicians and individuals at risk follow these symptoms and take the necessary precautions for the early detection of diabetes development. The study findings will contribute to the early diagnosis of diabetes using simple parameters and the prevention of complications that may develop due to the disease. It is of great importance for the society to know diabetes and related symptoms. In this context, studies on health literacy reveal that having information about diabetes improves individuals’ self-care activities [44]. According to the data used in our study, the average age of individuals with diabetes was found to be significantly higher (*p* < 0.05) and 69.6% of individuals aged 47 and over were diagnosed with diabetes positively. Increasing the level of knowledge of individuals about diabetes, monitoring the symptoms determined by association analysis, and encouraging them to consult a physician are of critical importance for early diagnosis, effectiveness of the treatment process, and prevention of complications.

In this study, it is also seen that the apriori algorithm can be used as a feature selection method. Feature selection is a widely used method in machine learning studies [16, 23], and offers various advantages such as reducing the size of the data set, increasing the speed of analysis, eliminating unrelated data, improving the quality of the data set and increasing the success of the results. In data mining studies for diagnosis and diagnostic processes, the use of machine learning methods and the apriori algorithm together has the potential to increase the reliability and accuracy of the results to be obtained.

Although it is seen in the literature that association analysis is widely used in areas such as transportation, traffic accidents, airlines, education, marketing, market basket analysis, maritime and environment [45–50], it is evaluated that its use in the health field is limited. This powerful method, which reveals the association structure of risk factors, can provide significant conveniences and innovations by being applied more widely in the health field. It is also important that such methods are adopted more in the health sector in order to achieve the “Health and Quality Life” target, which is among the Sustainable Development Goals.

The main limitation of this study is that the analyses were performed with data obtained from a single region. This methodology, which does not require blood test values, can be tested with more comprehensive data sets collected from different regions to evaluate whether symptoms show regional differences. In addition, the effect of association analysis on the success of machine learning methods in the diagnosis of diabetes will continue to be the subject of future studies.

## Conclusion

In conclusion, this study demonstrates the effectiveness of association analysis in identifying indicative symptoms for the early diagnosis of diabetes. The study findings indicate that the concurrent observation of eight symptoms (gender, polyuria, polydipsia, sudden weight loss, weakness, visual blurring, partial paresis, and obesity) has a significant impact on the development of diabetes. This method, which offers lower cost and faster results compared to traditional diagnostic methods, can contribute to the early detection of diabetes, accelerate treatment, and prevent potential complications. Association analysis, which is used in various fields in the literature, has received limited research in the healthcare field. However, the findings of this study highlight its potential for use in identifying chronic diseases such as diabetes. Replication of the study with larger datasets from different regions, analysis of regional differences in the identified symptoms, and more comprehensive investigation of the integration of association analysis with machine learning methods will provide important groundwork for further research.

## Data Availability

The dataset used in the study can be accessed in the material method section via reference number [29]. The relevant source has been cited.

## Declarations

### Relevant guidelines and regulations

All methods were carried out in accordance with relevant guidelines and regulations.
